# Myeloid-derived suppressor cells in the patients with liver resection for hepatitis B virus-related hepatocellular carcinoma

**DOI:** 10.1038/s41598-019-38785-3

**Published:** 2019-02-19

**Authors:** Wei-Chen Lee, Yu-Chao Wang, Chih-Hsien Cheng, Tsung-Han Wu, Chen-Fang Lee, Ting-Jung Wu, Hong-Shiue Chou, Kun-Ming Chan

**Affiliations:** 1 Division of Liver and Transplantation Surgery, Department of General Surgery, Chang-Gung Memorial Hospital, Linkou, Taiwan; 2 Chang-Gung University College of Medicine, Taoyuan, Taiwan

## Abstract

Liver resection remains the popular treatment for hepatocellular carcinoma (HCC). The aim of this study was to explore the alteration of immune cells in HCC patients with liver resections. Nineteen patients were included and their peripheral blood samples were taken before and after liver resections for immune-cell analysis. The clinical characteristics showed that the median diameter of the resected tumors was 7.5 cm with a range from 1.4 to 16.5 cm. The analysis of immune cells showed that the percentage of CD4^+^ T-cells were not altered by liver resection, but the percentage of CD8^+^ T-cell was decreased from 31.7 ± 12.4% to 20.2 ± 10.4% at one week after liver resection (p = 0.006). For immunosuppressor cells, regulatory T-cells were not altered, but myeloid-derived suppressor cells (MDSC) were decreased from 7.75 ± 8.16% to 1.51 ± 1.32% at one month after liver resection (p = 0.022) in 10 of 19 patients with high frequency of MDSC. Furthermore, it was also found that MDSC population was linearly correlated to tumor volume. In conclusion, CD8+ T-cell**s** and MDSC were altered by liver resection. The percentage of CD8+ T-cell**s** was decreased by surgery, but the accumulation of MDSC was abrogated.

## Introduction

Hepatocellular carcinoma (HCC) is an aggressive malignancy and one of the leading causes of cancer death. Several therapeutic options have been applied to treat HCC according to different stages. These treatment options includes surgical resection, liver transplantation, local ablation, transcatheter arterial chemoembolization (TACE) and systemic treatments^1–5^. Because liver transplantation is limited by lack of liver donors, liver resection is still the most popular option of the treatments for early stage HCC^6^. However, the treatment results are still not satisfactory because the tumors are easy to recur after liver resection^7^.

Immunity is the most important protection system for a host to defend cancer development. Cancer occurs as a consequence of enhanced or aberrant expression of oncogenes or loss of tumor suppressor genes. Cancer cells may express new tumor-specific or tumor-associated antigens which may be recognized by antigen-presenting cells and trigger T-lymphocyte-mediated anti-cancer immunity^8,9^. However, the hosts’ immunity can not function promptly in most of cancer patients who already have clinically diagnosed-cancers. In the patients with advanced cancer, the immune system may be further suppressed. Therefore, understanding of the immune suppression in cancer patients is essential for successful treatment of cancer.

Regulatory T cells and myeloid-derived suppressor cells (MDSC) are both immunosuppressive cells and stay with cancers^10,11^. Regulatory T cells were known to increase in the peripheral blood in HCC, gastric cancer, esophageal cancer, breast cancer and lung cancer^11–15^. Myeloid-derived suppressor cells (MDSC) are a population of cells of myeloid origin, including myeloid progenitors, immature macrophages, immature granulocytes and immature dendritic cells and characterized by production of reactive oxygen, nitrogen species and arginase I to suppress immunity^10,16^. In mice, CD11b^+^Gr-1^+^ MDSC cells inhibited T-cell responses by a NO-dependent mechanism and caused CD8^+^ T-cell apoptosis. In human, MDSC is identified as HLA-DR^−^CD33^+^ and its function is similar to that of mouse MDSC^10^. However, the role of MDSC in HCC was still limited^17–21^. This study focused on MDSC in HCC patients who received liver resection to remove the tumors.

## Results

### Patients

Seventeen male and 2 female patients who had liver resections for HCC were included in this study. The median (interquartile, IQ) age of these patients was 57 (44–66) years with a range from 34 to 70 years. Seventeen patients had hepatitis B and other 2 patients did not have viral hepatitis. Nine patients had cirrhosis and all were in Child-Pugh A classification. Eighteen patients had solitary tumor in the liver and only one patients had two tumors. The median (IQ) diameter of the tumors was 7.5 (3.4–11.2) cm with a range from 1.4 to 16.5 cm. By calculation, the median (IQ) tumor volume was 118 (18–354) cm^3^ with a range from 1.23 to 2008.5 cm^3^. All these patients had liver resections to remove the tumors completely. The operations and pre-operative clinical data were listed in Table 1.

**Table 1:** Clinical characteristics of 19 HCC patients with liver resection.

		Median (interquartile)(range)
Gender (M/F)	17/2	
Age (years)		57 (44–66) (34–70)
Hepatitis B (yes/no)	17/2	
Cirrhosis (yes/no)	9/10	
Operation		
Segmentectomy	10	
Left lobectomy	1	
Extended left lobectomy	1	
Right lobectomy	6	
Extended right lobectomy	1	
Tumor largest diameter (cm)		7.5 (3.4–11.2) (1.4–16.5)
Tumor volume (cm^3^)		118 (18–354) (1.23–2008.5)
α-fetoprotein (ng/ml)		45.2 (6.4–3352) (2–150806)
AST (IU/L)		55 (18–85) (23–101)
ALT (IU/L)		47 (18–85) (12–141)

AST, aspartate aminotransferase; ALT: alanine aminotransferase.

### CD4^+^ and CD8^+^ T-lymphocytes

**Figure 1:**
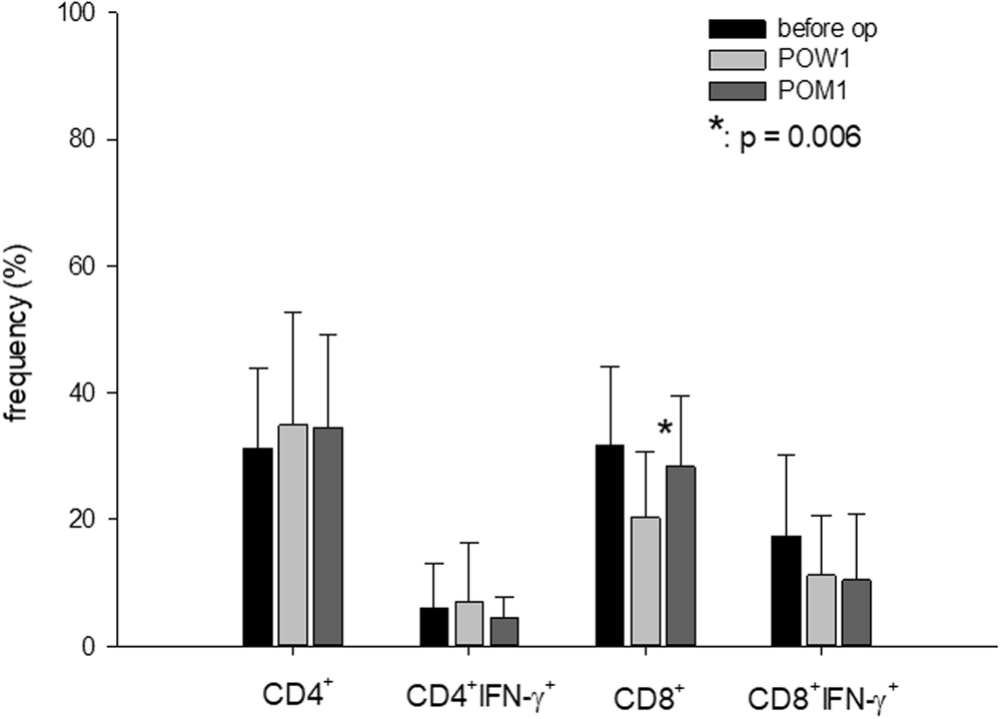
The frequency of immune cells in peripheral blood before and after liver resection. The frequency of CD4^+^ and CD4^+^IFN-γ^+^ were not altered by liver resection (p = 0.819 and 0.359, respectively). The CD8^+^ T-cell was 31.7 ± 12.4% before operation, decreased to 20.2 ± 10.4% at post-operation week one (POW 1) (p = 0.006) and returned to 28.3 ± 11.3% at one post-operation month one (POM 1). The CD8^+^ IFN-γ^+^ T-cell was 17.3 ± 12.8% before operation, decreased to 11.1 ± 9.5% at POW 1 and 10.4 ± 10.0% at POM1 (p = 0.105).

T-lymphocytes are the most important cells to mediate cellular immunity to cancer cells. To study the alteration of T-lymphocytes before and after liver resection, PBMC was stained with surface CD4 and CD8 molecules, and doubly stained with surface CD4 and CD8 molecules and intracellular cytokine interferon-γ (IFN) to determine the frequency of CD4^+^, CD4^+^IFN-γ^+^, CD8^+^ and CD8^+^IFN-γ^+^ cells. The results showed that frequency of CD4^+^ and CD4^+^IFN-γ^+^ were not altered by liver resection (p = 0.819 and 0.359, respectively). The frequency of CD8^+^ T-cell was 31.7 ± 12.4% before liver resection, decreased to 20.2 ± 10.4% at one week after liver resection (p = 0.006) and returned to 28.3 ± 11.3% at one month after liver resection. The frequency of CD8^+^ IFN-γ^+^ T-cell was 17.3 ± 12.8% before liver resection, decreased to 11.1 ± 9.5% at one week after liver resection and 10.4 ± 10.0% at one month after liver resection. There was a trend of CD8^+^ IFN-γ^+^ T-cell decrease after liver resection (p = 0.105) (Fig. 1).

### Immunosuppressor cells

**Figure 2:**
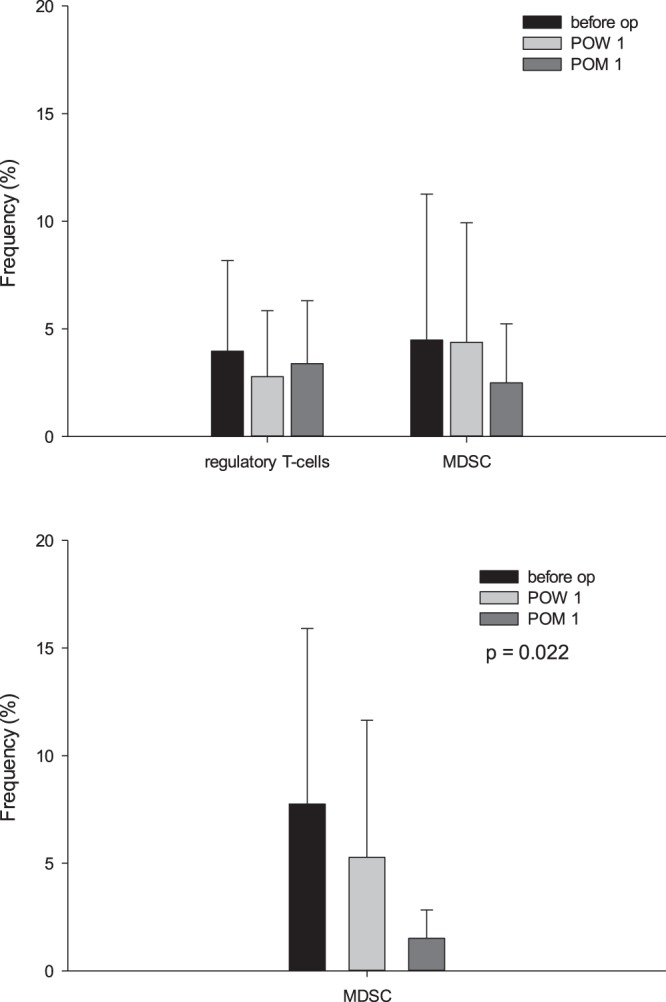
The frequency of regulatory T-cells and MDSC in peripheral blood before and after liver resection. (**a**) The frequency of regulatory T-cells was 2.26 (0.67–6.93)% before liver resection, 1.95 (0.28–4.16)% at one week after liver resection and 2.64 (1.49–4.59)% at one month after liver resection (p = 0.165). The frequency of MDSC was not significantly changed before or after liver resection, either. The median (IQ) frequency of MDSC was 2.40 (0.96–5.65) % before liver resection, compared to 1.93 (0.59–5.54)% at one week after liver resection and 1.44 (0.70–3.50)% at one month after liver resection (p = 0.549). (**b**) Among 19 patients, 10 patients had MDSC >2% before liver resection. For these 10 patients, the frequency of MDSC was 7.75 ± 8.16% before operation, and decreased to 5.27 ± 6.37% at POW 1 and further to 1.51 ± 1.32% at POM 1 (p = 0.022).

Regulatory T-cells and MDSC are the well-known immunosuppressor cells. The frequency of regulatory T-cells and MDSC were determined before and after liver resection. The results showed that the median (IQ) frequency of regulatory T-cells was 2.26 (0.67–6.93)% before liver resection, 1.95 (0.28–4.16)% at one week after liver resection and 2.64 (1.49–4.59)% at one month after liver resection (p = 0.165). The frequency of MDSC was not significantly changed before or after liver resection, either. The median (IQ) frequency of MDSC was 2.40 (0.96–5.65)% before liver resection, compared to 1.93 (0.59–5.54)% at one week after liver resection and 1.44 (0.70–3.50)% at one month after liver resection (p = 0.549) (Fig. 2a). However, if we focused on the 10 of 19 patients with MDSC >2% before liver resection, the frequency of MDSC was 7.75 ± 8.16% before liver resection, and decreased to 5.27 ± 6.37% at one week and further to 1.51 ± 1.32% at one month after liver resection (p = 0.022, Fig. 2b).

### Correlation between tumor sizes and MDSC

**Figure 3:**
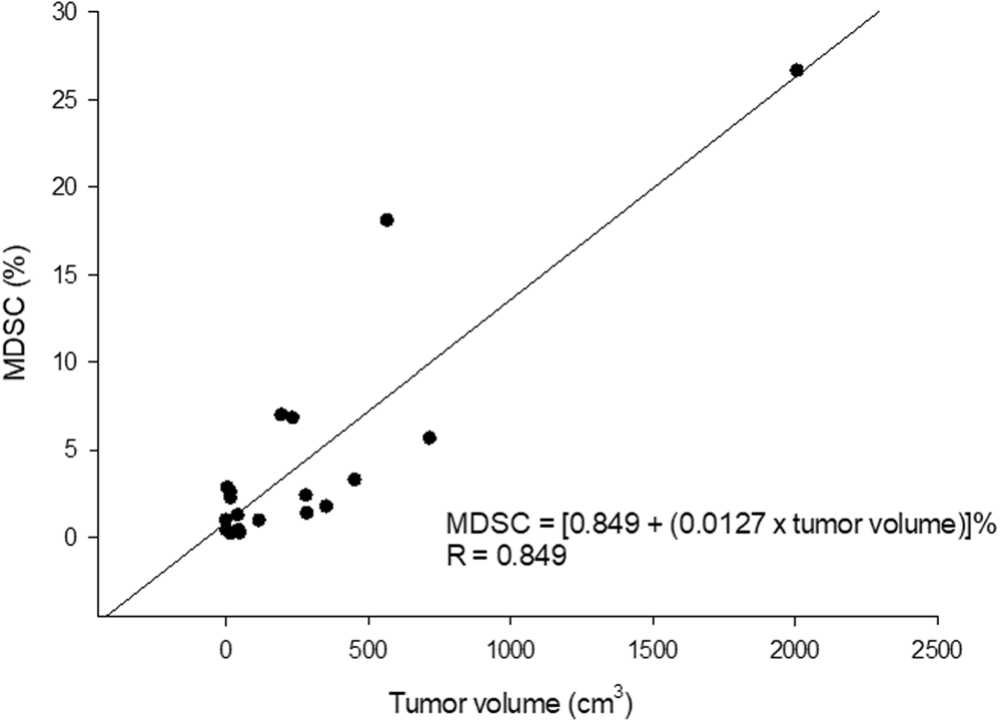
The relationship between MDSC and tumor volume. The frequency of MDSC in peripheral blood was linearly regressed to tumor volume. The frequency of MDSC was equal to [0.849 + (0.0127 × tumor volume (cm^3^))]%.

The frequency of MDSC was most likely expanded in the patients with large-sized HCC. To determine whether the population of MDSC was correlated to tumor sizes, regression analysis between MDSC and tumor sizes was performed. The result showed that the frequency of MDSC in peripheral blood was linearly regressed to tumor volume. The equation was frequency of MDSC = [0.849 + (0.0127 × tumor volume (cm^3^))]% (r = 0.876, Fig. 3).

### Postoperative survival

**Figure 4:**
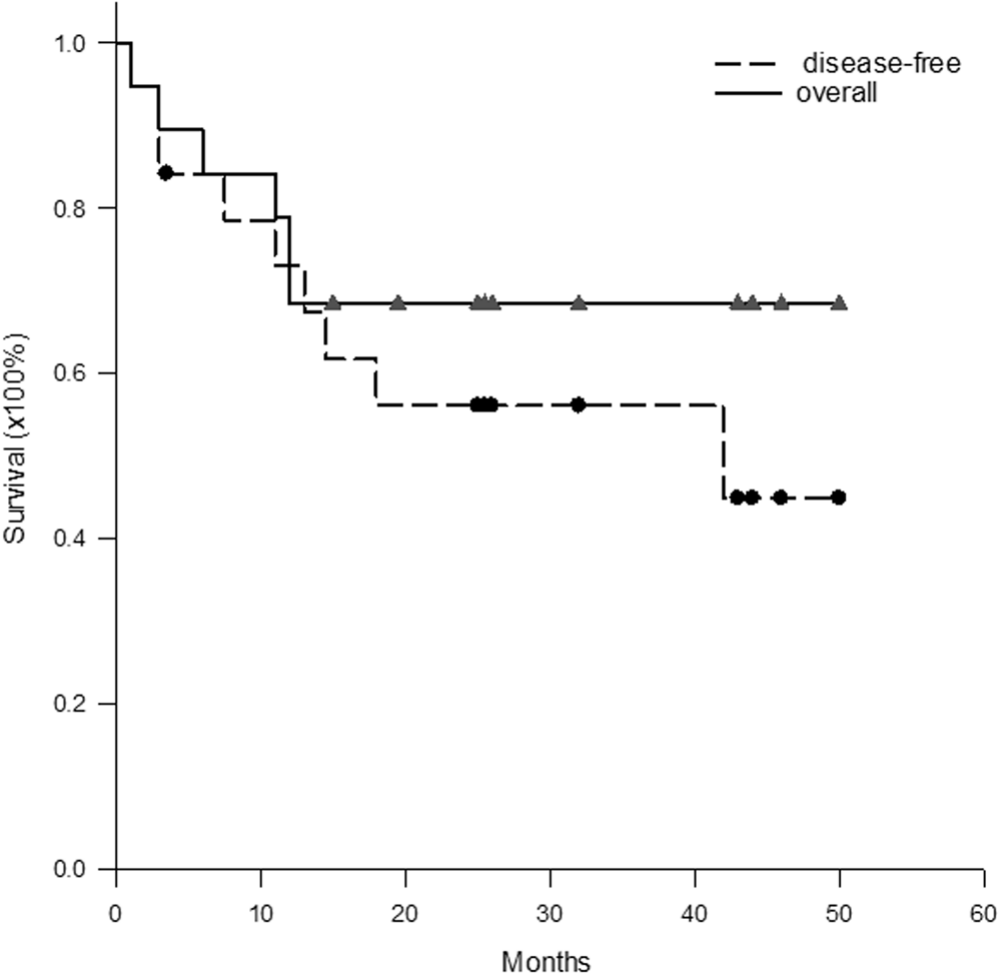
The disease-free and overall Kaplan-Meier survival curve. The 1- and 3-year disease-free survival rates were 73.0% and 56.1%, and overall survival rates were 78.9% and 68.4%, respectively, for all the patients.

After operations, all patients were discharged from the hospital, however, one patient died of myocardiac failure at one month after liver resection. All the other patients were regularly followed up at out-patient clinic. The mean follow up was 25.8 ± 16.4 months. Nine patients had tumor recurrence until now. The 1- and 3-year disease-free survival rates were 73.0% and 56.1%, respectively. The 1- and 3-year overall survival rates were 78.9% and 68.4%, respectively (Fig. 4).

### Survival rates according to tumor volume

**Figure 5:**
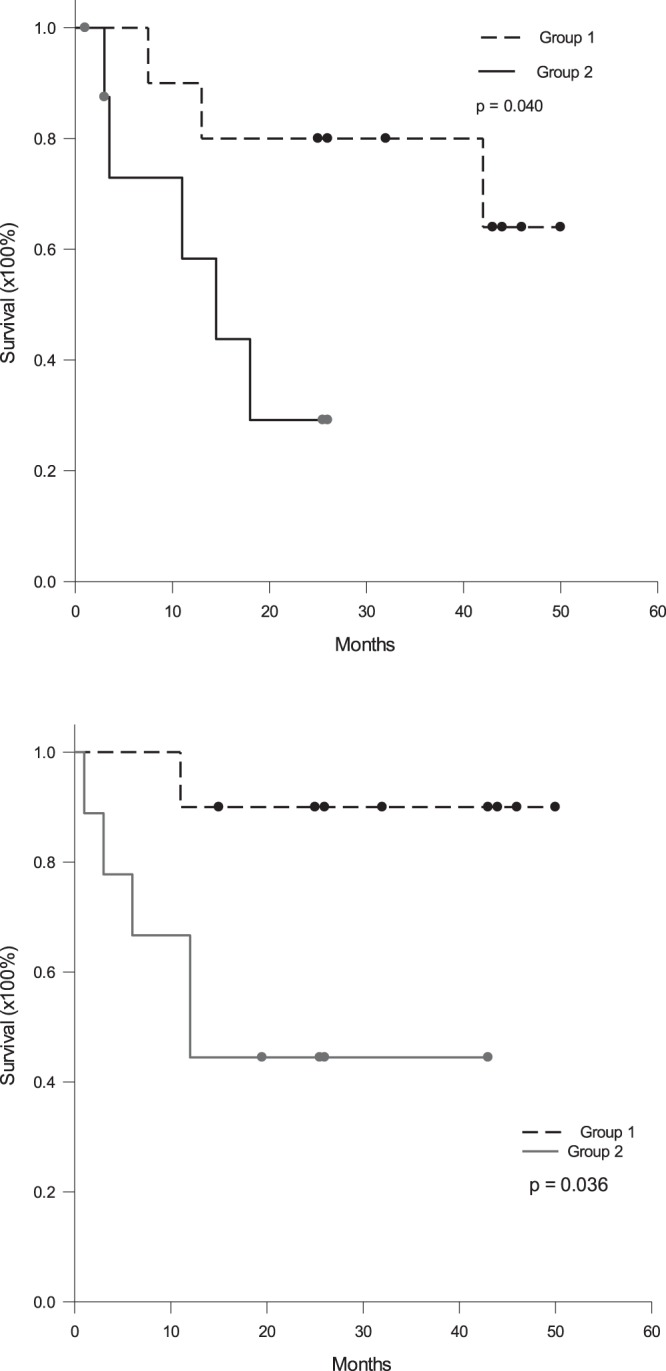
Disease-free and overall survival rates according to tumor volume. The patients were divided into two groups according to median tumor volume: group 1 patients with tumors ≤118 cm^3^ and group 2 patients with tumors >118 cm^3^. (**a**) The 1- and 3-year disease-free survival was 90.0% and 90.0% for group 1 patients, compared to 58.3% and 29.2% for group 2 patients (p = 0.040). (**b**) The 1- and 3-year overall survival was 90.0% and 80.0% for group 1 patients, compared to 66.7% and 44.4% for group 2 patients (p = 0.036).

To determine whether the survival rates were different in different tumor sizes, the patients were divided into two groups according to median tumor volume: group 1 patients with tumors ≤118 cm^3^ and group 2 patients with tumors >118 cm^3^. The median (IQ) frequency of MDSC was 0.97 (0.36–2.33)% in group 1 patients, compared to 5.65 (2.07–12.54)% in group 2 patients (p = 0.003). The 1- and 3-year disease-free survival was 90.0% and 90.0% for group 1 patients, compared to 58.3% and 29.2% for group 2 patients (p = 0.040, Fig. 5a). The 1- and 3-year overall survival was 90.0% and 80.0% for group 1 patients, compared to 66.7% and 44.4% for group 2 patients (p = 0.036, Fig. 5b).

### Correlation between tumor sizes and AFP

**Figure 6:**
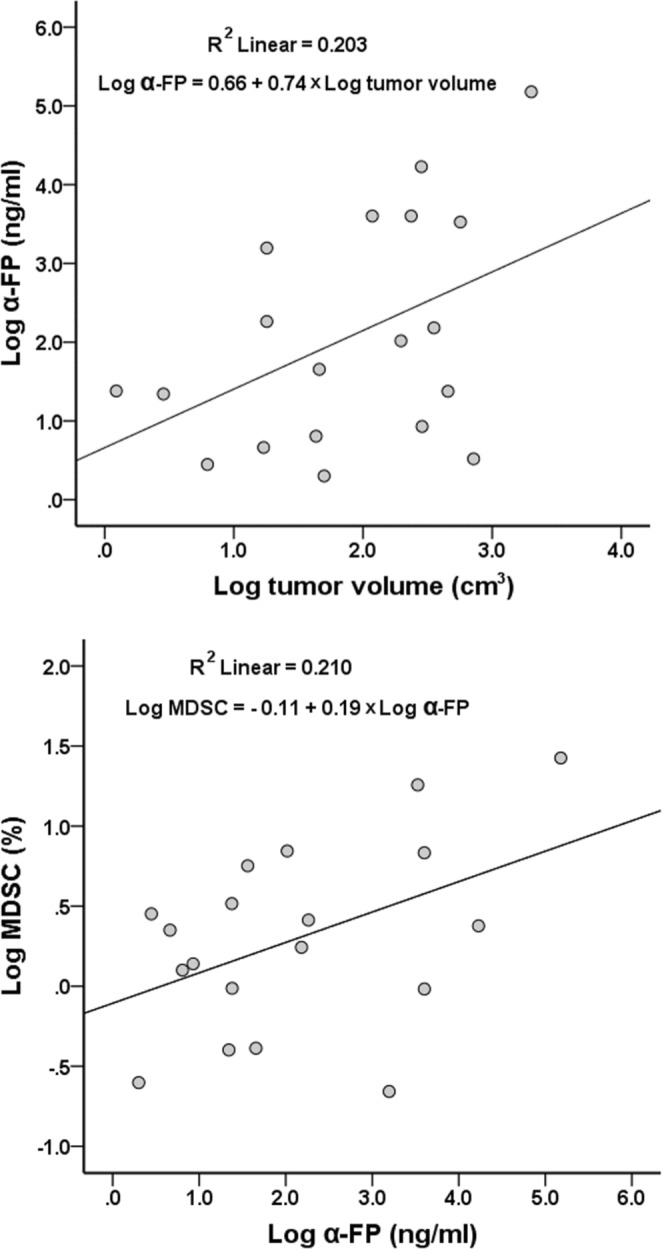
The correlation between AFP to tumor volume and frequency of MDSC. (**a**) The serum level of α-FP has moderately positive correlation to tumor volume. The equation is logAFP = 0.66 + 0.74 × log tumor volume (r = 0.451, p = 0.053). (**b**) The frequency of MDSC in peripheral blood has moderately positive correlation to AFP. The equation was logMDSC = −0.11 + 0.19 × logAFP (r = 0.458, p = 0.049).

AFP is a biomarker of HCC and is related to tumor biology. The higher level is AFP, the worse is the prognosis. To determine whether the serum levels of AFP were correlated to tumor volume, regression analysis between AFP and tumor volume was performed. Because the value of AFP and tumor volume varied greatly, log transformation of AFP and tumor volume was performed to undergo Pearson’s correlation test. The result showed that the serum level of AFP had moderately positive correlation to tumor volume. The equation was log AFP (ng/ml) = 0.66 + 0.74 × log tumor volume (cm^3^) (r = 0.451, Fig. 6a).

### Correlation between AFP and MDSC

Because both frequency of MDSC and serum levels of AFP were correlated to tumor volume, frequency of MDSC and serum levels of AFP may be related closely. Because the value of AFP and MDSC varied greatly, log transformation of AFP and MDSC was performed to undergo Pearson’s correlation test. The result showed that the frequency of MDSC in peripheral blood had moderately positive correlation to AFP. The equation was log MDSC = −0.11 + 0.19 × log AFP (ng/ml)) (r = 0.458, Fig. 6b).

## Discussion

MDSC is an immunosuppressive cell and attracts attention for cancer and chronic diseases in a recent decade. This study showed that the frequency of MDSC in peripheral blood of HCC patients was linearly correlated to tumor volume and could be calculated by a formula using tumor volume. It was known in murine study that MDSC appeared in the mice bearing large tumors^22,23^. Dr. Kapanadze *et al*. reported that MDSC appeared at late stage of carcinogenesis in murine HCC model which mimicked human diseases^17^. Clinically, it was reported that the population of MDSC in PBMC of HCC patients was much higher than in healthy normal control and non-tumor cirrhotic patients^20,24^. This study showed that the frequency of MDSC in peripheral blood could be quantitatively estimated according to the equation between the frequency of MDSC and tumor volumes.

The frequency of MDSC could be reduced by excision of tumor. When we focused on the patients with large tumors and high frequency of MDSC, the frequency of MDSC was reduced to normal level at one month after liver resection. In animal study, the population of MDSC was reduced after the tumor was removed and the survival of the mice was prolonged^25^. Dr. Gabrilovich described that granulocyte colony-stimulating factor, macrophage colony-stimulating factor, granulocyte/macrophage colony-stimulating factor), stem cell factor, vascular endothelial growth factor and other cytokines might be the factors to expand MDSC^10^. As the frequency was expanded by tumor, some of these cytokines might be released by tumors. Excision of HCC became the most direct way to block the stimulatory factors and returned the frequency of MDSC back normal.

The disease-free and overall survival of the patients with large tumors were much worse than those patients with small-sized tumors. Tumor size is a well-known risk factor for patients with liver resection for HCC^7^. In this study, when the patients were divided into two groups according median tumor volume 118 cm^3^, the patients with tumor volume >118 cm^3^ had much worse disease-free and overall survival rates than the patients with tumor volume ≤118 cm^3^. As the results of population of MDSC directly related to tumor sizes in this study, the patients with large-sized tumors had high population of MDSC and low anti-tumor immunity. Subsequently, the patients with large tumors had higher incidence of tumor recurrence and worse postoperative survival. When the tumors were resected to decrease tumor burden, the population of MDSC was decreased but the low anti-tumor immunity could not be reversed completely. Although many factors were well-known and contributed to worse prognosis after liver resection for HCC, the high frequency of MDSC might be one of them, but rarely be mentioned.

T-lymphocytes are the direct effector cells to attack cancer cells, particularly CD8^+^ T-cells. Helper CD4^+^ T-cells were not altered before and after liver resection, however, CD8^+^ T-cells were significantly decreased at one week after liver resection and recovered at one month. It was clearly show that surgery itself was immunosuppressive. When we looked at CD8^+^IFN-γ^+^ T-cells, this effective cytotoxic CD8^+^IFN-γ^+^ T-cells had the tendency to decrease after liver resection and did not recover at one month after liver resection. It implied that both tumor and surgery were immunosuppressive. Hosts’ immunity was not fully recovered although the tumors were totally removed.

Although only two-third of HCC patients have elevated serum level of AFP, AFP is recognized as a biomarker of HCC. In this study, serum AFP level had moderately positive correlation to tumor volume and MDSC. High level of AFP was a poor prognostic factor. Dr. Peng *et al*. mentioned that high level of AFP was associated with lower 10-year survival rate, particular in large tumors^26^. AFP is also recognized as a marker of biology of HCC, particular in liver transplantation setting. In liver transplantation, AFP is a risk factor of tumor recurrence after liver transplantation and high level of AFP is even not suitable to undergo liver transplantation^27,28^. Obviously, AFP level reflects aggressive biology of HCC with poor prognosis even after the tumors were removed.

Regulatory T-cells are another immunosuppressive cells. Regulatory T-cells were not changed before and after liver resection in this study. Hoechst *et al*. reported that regulatory T-cells were induced by MDSC when autologous T-cells were co-cultured with MDSC^20^. In our previous report, we found that the frequency of regulatory T-cells was higher the in tumor tissues of large tumors than small tumors, but was not different in peripheral blood^12^. In this study, we had the similar data that the frequency of regulatory T-cells in peripheral blood was not related to MDSC linearly. It did not show direct relationship between regulatory T-cells and tumor volume in this study, either.

The limitation of this study was the limited number of patients. Because the number of the patients were limited, some significant changes before and after liver resection could not be demonstrated. However, the population of MDSC and serum level of AFP correlated to tumor volume still could be well-expressed.

In conclusion, the frequency of MDSC was well correlated to tumor volume of HCC. Removal of tumors could reduce the population of MDSC. However, the remained unsatisfied prognosis of the patients with large tumor implied the low immunity of the patients with large tumors would not recover completely after tumor resection. To improve prognosis, enhancement of anti-tumor immunity has to be performed after surgery. Further studies are needed to meet the unmet.

## Methods and Materials

### Patients

Nineteen patients, 17males and 2 females, who received liver resection for early stage HCC from Dec. 2013 to Nov. 2016 were included in this study. All the patients signed informed consent to joint this clinical study. After informed consent was obtained, 10 cc of peripheral blood sample was withdrawn before liver resection, and one week and one month after liver resection for immune cell analysis. Clinical characteristics of the patients including age, gender, liver function and white blood cell count were all recorded. This study protocol confirmed to the ethical guidelines of the 1975 Declaration of Helsinki and was approved by institutional review board of Chang-Gung Memorial Hospital (IRB No. 101-3552B).

### Immune cells identification

Peripheral blood monocytes (PBMC) were isolated from peripheral blood by Ficoll-Hypaque (GE Healthcare, Uppsala, Sweden) density centrifugation. The phenotypes of immune cells in the peripheral blood were identified by flow cytometry after stained with fluorescence-conjugated monoclonal antibodies. The surface monoclonal antibodies included anti-CD4 (RPA-T4 clone; PharMingen, San Diego, CA), anti-CD8 (RPA-T8 clone; PharMingen), anti-CD33 and anti-HLA-DR. The intracellular foxp3 was stained by fluorescence-conjugated rat anti-human foxp3 (eBioscience,San Diego, CA). The expression of these molecules was analyzed by cytofluorography employing a Beckman Coulter NAVIOS flow cytometer (Beckman Coulter Co., Indianapolis, IN). Regulatory T-cells were identified as positive for CD4 and foxp3. MDSC was identified as positive for CD33 and negative for HLA-DR.

### Intracellular cytokine staining

Brefeldin A (5 ug/ml) was added into the *in vitro* culture of PBMC for 4 hours. The cell was fixed by 2% paraformaldehyde and permeabilized by saponin (0.5%). Intracellular cytokine was analyzed by cytofluorography employing a Beckman Coulter NAVIOS flow cytometer (Beckman Coulter Co., Indianapolis, IN) after the intracellular cytokine was stained by PE-conjugated mouse anti-human IFN-γ (1/50x; PharMingen, San Diego, CA).

### Operation technique

All operations were performed by open methods. During operation, intra-operative ultrasonography was performed first to demarcate the tumor and identified the relationship between tumors and major vessels. Intermittent Pringle’s maneuver, 15-minute clamping followed by 5-minute release, was applied for most of the operations. Parenchymal transection was performed by ultrasonic dissector (CUSA, Valley-lab, Inc, Boulder, CO).

### Characteristics of tumors

The final diagnosis of HCC was based on pathological reports. Tumor sizes were documented as those were measured during gross-examination by pathologists. The tumor volume was calculated by the formula: tumor volume = 0.52 × width^2^ × length^29^. The microscopic examination of tumor cell differentiation, encapsulation of tumors and microvascular invasion were all recorded.

### Follow up

The patients were followed up regularly after liver resection. Tests of liver function and α-fetoprotein, and liver ultrasonography were performed every 3 months. Dynamic computed tomography (CT) of the liver was performed if deemed necessary. Tumor recurrence was defined when CT detected tumors with typical HCC imaging pattern in the liver or extrahepatic tumors. Disease-free survival was measured from the date of surgical treatments to tumor recurrence. Overall survival was measured from the date of surgical treatments to date of last following up or patients’ death. Hospital mortality was defined that patients were not discharged and died in hospital after surgery.

### Biostatistics analysis

The comparisons of categorical variables were determined by Chi-square Tests. The significance of the differences between different groups was determined by unpaired or paired Student’s t-test. The disease-free and overall survival rates were calculated using the Kaplan-Meier method and compared between groups using the log-rank test. While the data of MDSC or α-FP varied greatly, Log transformation of MDSC or α-FP was performed to pass Shapiro-Wilk test. The relationship between tumor volume and MDSC or α-FP was performed by Pearson’s correlation. The statistical analyses were all performed with SigmaPlot 12.3 software for Windows (Systat Softwave, Inc., San Jose, CA, USA). P value below 0.05 was considered to be significantly different.
